# Technology-Assisted Rehabilitation of Writing Skills in Parkinson's Disease: Visual Cueing versus Intelligent Feedback

**DOI:** 10.1155/2017/9198037

**Published:** 2017-10-08

**Authors:** Evelien Nackaerts, Alice Nieuwboer, Elisabetta Farella

**Affiliations:** ^1^Neuromotor Rehabilitation Research Group, Department of Rehabilitation Sciences, KU Leuven, Leuven, Belgium; ^2^E3DA Research Unit, ICT Center, Fondazione Bruno Kessler, Trento, Italy

## Abstract

Recent research showed that visual cueing can have both beneficial and detrimental effects on handwriting of patients with Parkinson's disease (PD) and healthy controls depending on the circumstances. Hence, using other sensory modalities to deliver cueing or feedback may be a valuable alternative. Therefore, the current study compared the effects of short-term training with either continuous visual cues or intermittent intelligent verbal feedback. Ten PD patients and nine healthy controls were randomly assigned to one of these training modes. To assess transfer of learning, writing performance was assessed in the absence of cueing and feedback on both trained and untrained writing sequences. The feedback pen and a touch-sensitive writing tablet were used for testing. Both training types resulted in improved writing amplitudes for the trained and untrained sequences. In conclusion, these results suggest that the feedback pen is a valuable tool to implement writing training in a tailor-made fashion for people with PD. Future studies should include larger sample sizes and different subgroups of PD for long-term training with the feedback pen.

## 1. Introduction

Parkinson's disease (PD) is a neurodegenerative disorder characterized by the loss of dopaminergic neurons in the basal ganglia leading to a combination of motor and nonmotor symptoms [1]. In addition to the primary symptoms, that is, tremor, rigidity, bradykinesia, and postural instability, micrographia is a frequently occurring problem [1]. Micrographia is defined as “an impairment of a fine motor skill manifesting mainly as a progressive reduction in amplitude during a writing task” [2]. For treatment of PD, dopaminergic medication is the gold standard, though not all symptoms respond equally well [3]. Therefore, motor rehabilitation is often a necessary therapeutic supplement [4]. Several studies have shown that motor learning is possible in PD, although learning occurs more slowly and with less automaticity (for reviews see [5, 6]). Motor performance and the learning potential in PD can be further improved by means of cueing and feedback strategies [7, 8]. Cues are defined as a reference or trigger for movement generation [9]. Feedback refers to the provision of external information which supplements the internal sensory pathways to guide learning online or after performance [10]. The beneficial effects of both types of input are often attributed to the fact that they induce a shift in motor control from a habitual to a goal-directed modus or, in other words, induce redirection from more to less affected neural circuits [11].

The benefits of cueing and feedback have mainly been shown for gait in PD [12, 13]. Unlike gait, handwriting incorporates both automated and controlled processes [14]. As such, cueing and feedback strategies may have an alternate effect. Several studies have shown benefits of short-term training with visual cues [15, 16]. However, recently, it was shown that visual cues sometimes hamper handwriting, especially when cueing smaller writing sizes, as such introducing an additional accuracy constraint [17]. As a result, the visual system may have become overloaded, increasing difficulty [18]. Hence, using other sensory modalities to deliver cueing or feedback should be considered. Providing supplementary sensory information may aid motor learning as well as motor performance [19]. In contrast, when cueing or feedback was removed, motor performance worsened in PD [20, 21]. Similarly, when feedback was provided too frequently during motor learning, that is, in 100% of the trials, it caused dependency and lack of transfer [22, 23]. To counteract this drawback, providing external input intermittently may be a valuable alternative. With recent technological advances, it is possible to realize individualized and intelligent feedback adjusted to performance outcomes [24–28]. To the best of our knowledge, this has not been studied for upper limb tasks such as writing in PD.

Therefore, an intelligent pen that can provide real-time feedback in an intermittent manner was developed to address micrographia. The current proof-of-concept study was designed to compare the effects of short-term training (one session) with continuous visual cues with intermittent intelligent feedback. We expected that training with the continuous visual cue would lead to more dependency and less improvement of writing amplitude compared to training with intelligent feedback.

## 2. Methods

### 2.1. Participants and Experimental Protocol

In this cross-sectional study, 10 patients with PD and 10 healthy controls (CT) were assessed for eligibility. Inclusion criteria for patients were (i) diagnosis of PD according to the UK Brain Bank Criteria [29]; (ii) Hoehn and Yahr (H&Y) stages I–III in the on-phase of the medication cycle [30]; and (iii) being on stable medication. Exclusion criteria for both groups were (i) cognitive impairment (Mini-Mental State Examination, MMSE < 24) [31] and (ii) interfering upper limb problems. As such, one healthy control was excluded from the analyses due to MMSE < 24.

Participants were randomly assigned to one of two training programs, that is, either continuous cueing with visual target zones (Cue), or (ii) intermittent intelligent feedback, that is, providing verbal corrections during writing when it deteriorated (Feedback). The session started with assessment of baseline writing performance. This was followed by a short training period using one of the two training methods. After the training session, writing performance was assessed again. In addition to the writing tests, disease-specific features were determined using the Movement Disorder Society Unified Parkinson's Disease Rating Scale (MDS-UPDRS) part III [32], New Freezing of Gait (NFOG) questionnaire [33], and Levodopa Equivalent Dose (LED) [34]. In addition, the Manual Ability Measure (MAM-16) [35] and Edinburgh Handedness Inventory [36] were completed by both patients and controls. For patients, testing of writing performance and disease-specific characteristics occurred during the on-phase of the medication cycle, that is, approximately 1 h after medication intake.

The study design and protocol were approved by the local Ethics Committee of the University Hospitals Leuven and were in accordance with the code of Ethics of the World Medical Association (Declaration of Helsinki, 1967). After explanation of the study protocol, written informed consent was obtained from all participants prior to participation in the study.

### 2.2. Writing Assessment

**Figure 1:**
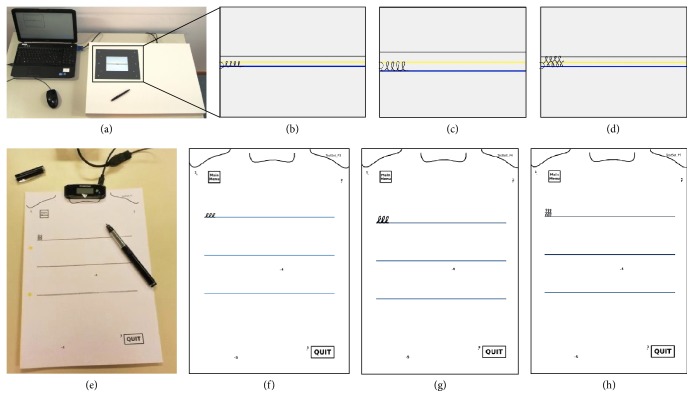
*Touch-sensitive tablet and feedback pen system*. (a) Setup of the tablet; (b–d) examples of the small trained (b), large trained (c), and untrained (d) task with visual cues. It has to be noted that testing was performed in the absence of the yellow (middle) and upper (grey) line. (e) Setup of the pen, receiver, and paper; (f–h) examples of the test sheets for the small trained (f), large trained, (g) and untrained task (h).

Writing performance was assessed before and after a short training period, both on a touch-sensitive writing tablet (Figure 1(a)) [37] and with a custom-made feedback pen (Figure 1(e)) in a counterbalanced order. Three exercises were performed: (i) writing of continuous loops, resembling the letter “e” (0.6 cm) (Figures 1(b) and 1(f)); (ii) writing of continuous loops, resembling the letter “l” (1.0 cm) (Figures 1(c) and 1(g)); and writing of a figure of 8-like movement (1.0 cm) (Figures 1(d) and 1(h)). Both continuous loops were practiced during the training period (i.e., trained tasks), while the figure of 8-like movement was not (i.e., untrained task) to study short-term transfer effects. All tests were previously used in studies using a touch-sensitive writing tablet [38, 39] and were performed in the absence of visual cueing and intelligent feedback to assess transfer.

### 2.3. Intervention

The training session lasted approximately 30 min including short breaks. All participants performed a minimum of eight and maximum of 12 writing exercises, depending on the subjective reporting of fatigue. Each exercise consisted of writing different types of preletters for a duration of 90 s. A training session included two exercises with the letter “e,” two exercises with the letter “l,” and four to eight exercises with alternative preletters (e.g., resembling the letter “v” or “n”). Training with continuous visual cueing was performed on the tablet. Visual cues consisted of colored visual target zones indicating the requested writing size, similar to the ones used in the study by Nackaerts et al. [17]. While in the latter study visual cues were merely offered, participants in the present study were encouraged to increase their amplitude using the cues. The intelligent feedback was provided using a newly developed feedback pen and exercises were performed on regular paper. Feedback was provided intermittently, that is, every 6 s, and consisted of one of five types of feedback messages depending on the writers' performance: (i) good; (ii) try to write larger; (iii) try to write smaller; (iv) try to write slower; and (v) try to write faster. As micrographia was the focus of this study, priority was given to feedback messages with respect to writing amplitude over writing speed. Subjects were instructed to attend to the feedback and alter their performance accordingly.

It is important to note that cued training was only performed on the tablet and not on paper, while feedback training was only performed on paper and not on the tablet.

### 2.4. System Design

The requirements of the feedback pen were as follows: (i) to accurately capture and process spatial and temporal coordinates of the written trace of a ball-point pen on a regular sheet of paper and (ii) to provide verbal information in real time on a specific writing feature (e.g., amplitude or speed). Therefore, a prototype was developed based on a commercially available digital pen, augmented with appropriate hardware and firmware. The final system consisted of a digital pen with a microcontroller-based add-on board, designed to enable feature extraction and audio feedback. We selected the Staedtler Digital Pen 990 (Staedtler Mars GmbH & Co., Nuremberg, Germany) for its characteristics in terms of working area (166 × 125 mm^2^), sample frequency (66 Hz), and accuracy (0,126 × 0,126 mm^2^/point) when used in pen mode. More detailed technical information is presented in Guardati et al., 2015 [40].

The final system consisted of three main parts. A first part is the preprinted paper, consisting of a specific exercise (examples in Figure 1). On each paper, possible locations for the receiver of the pen were included for convenience of the user. In addition, the paper also served as an interface, allowing interactive calibration. The second part consisted of the Staedtler Digital Pen and receiver. This receiver collected the coordinates and sent them via USB to the third part, that is, the add-on board. The add-on board was the novelty of the system, as it allows correct interpretation of data based on a calibration process and real-time processing of the writing features. The board was based on a Cortex M4 microcontroller, working at 168 Mhz, with 1024 Kb of flash memory and 192 Kb of RAM. It included an audio Codec, an SD card reader, and a loudspeaker. A real-time operative system, Nuttx RTOS (http://nuttx.org/), ran on the microcontroller providing a flexible and modular environment for easy development and debugging. Libraries for interfacing with the various parts of the system were implemented, in particular to communicate with the pen. On top of that, it ran the firmware based on FiMoSDK (Fine Movement Software Development Kit), a custom library in C++ that implements the handwriting exercises.

The first purpose of the board was to calibrate the system in order to avoid problems with the interpretation of data as a result of misalignment between the expected and actual position of the receiver on the paper. Therefore, the user was requested to put the pen at five calibration points by means of an audio-guided start-up. These points, watermarked on the paper (Figure 1), were compared with the “default” reference system that was determined in controlled conditions. This allowed a rototranslation to align the reference system. Although three points would be sufficient, we chose a redundancy approach to be able to discard up to two points in case of noise or errors during the acquisition. As such, requests for repeating the calibration to the user were minimized.

The second main functionality of the add-on board was to generate audio feedback. The system could be configured in three different modalities: (i) no feedback; (ii) continuous reminder (not included in the current study); and (iii) intelligent feedback. The first modality was used to assess handwriting without the provision of additional information. The continuous reminder was a periodic signal that did not depend on user performance and just reminded the subject to write in a certain manner (e.g., remember to write big). The intelligent feedback depended on the user performance, measured in real time. In this third modality, a certain feature, such as writing amplitude or speed, was detected while the user was writing and compared to a preset target performance value. The allowed deviation from this target needed to be defined in advance. Comparison between the target value and the actual performance determined the kind of feedback (e.g., “good” and “try to write larger”). Detailed specifications were explained in Guardati et al., 2015 [40].

### 2.5. Data Processing and Statistical Analysis

All data from the pen and tablet were filtered at 7 Hz with a 4th-order Butterworth filter and further processed using Matlab R2011b. Writing amplitude (cm) was determined by calculating the differences between the local minima and maxima of each individual stroke [37].

Statistical analysis was performed using SPSS (IBM SPSS statistics version 24). Normality of the data was assessed by means of the Shapiro-Wilk test. The Mann–Whitney *U* and the Pearson Chi Square test were used to compare differences in demographic characteristics between both training types. Paired *t*-tests were used to look for systematic differences between writing performance on the tablet and with the pen. To investigate the effect of training, a repeated measures analysis of variance (ANOVA) was performed, with training type (Cue versus Feedback) and group (PD versus CT) as between-subject factors and time (pre versus post) as a within-subject factor. This analysis was performed for the three tasks separately with both measurement tools. Effect sizes were measured by means of the partial eta-squared.

### 2.6. Feasibility and User Satisfaction

At the end of the session, all patients filled out a questionnaire on how much they wrote in daily life and whether they were familiar with the use of a laptop, tablet, or smartphone. In addition, they were asked whether they were interested in training with the system at home, if so how frequently and whether they had suggestions for improvement.

## 3. Results

### 3.1. General Characteristics and Tool Comparison

General group characteristics did not differ significantly between training types (Table 1). Additionally, there was no significant difference in the amount of exercises performed during training (*t* = 0.980, *p* = 0.341).

For writing at the smaller size (letter “e”), no differences were found between writing with the pen and on the tablet (*t* = 0.450, *p* = 0.659). For the larger writing sizes, a systematic difference was found for the letter “l” (*t* = 4.148, *p* = 0.001) and to a lesser extent for the figure of 8-like movement (*t* = 1.849, *p* = 0.082), showing that writing amplitude with the pen was smaller than when assessed with the tablet.

### 3.2. The Effect of Training on Writing Amplitude

**Figure 2:**
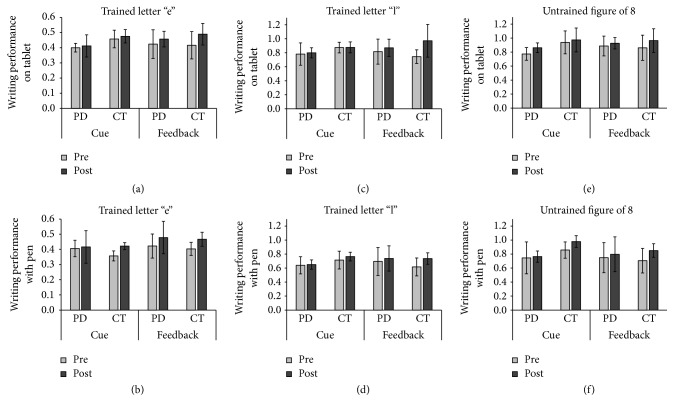
*The effects of short-term training with visual cues or intelligent feedback*. Results are displayed for the different tasks and groups, performed both on the touch-sensitive writing tablet and with the feedback pen.

For the test with the trained letter “e,” main effects of time were found during both writing tests on the tablet (*F* = 3.461, *p* = 0.083; *η*^2^ = 0.187) and writing tests with the pen (*F* = 6.692, *p* = 0.023; *η*^2^ = 0.340). Although the former only revealed a tendency, both test methods exposed an increased writing amplitude after training regardless of the training type (Figures 2(a) and 2(b)). For the trained letter “l,” a main effect of time was found for writing assessed on the tablet only (*F* = 5.423, *p* = 0.034; *η*^2^ = 0.266), showing a larger amplitude after training. However, there was also a strong trend towards an interaction between training type and time in this condition (*F* = 3.975, *p* = 0.065; *η*^2^ = 0.209). Exploratory post hoc analysis revealed that only the group that received feedback training improved significantly from baseline to posttraining (*p* = 0.036, Bonferroni-corrected) (Figure 2(c)). Finally, main effects of time were found for the untrained task and this for both writing on the tablet (*F* = 7.129, *p* = 0.017; *η*^2^ = 0.322) and writing with the pen (*F* = 6.470, *p* = 0.026; *η*^2^ = 0.350). Both displayed an increase in amplitude from baseline to posttraining (Figures 2(e) and 2(f)).

### 3.3. Feasibility and User Satisfaction

All patients were computer-literate and five were also employing a tablet or smartphone. Across training types, patients were interested in a long-term training program at home, if the exercises would not only include preletters but would become gradually more difficult. Two patients reported problems with the grip of the pen, one in the cue and one in the feedback group. Furthermore, the calibration of the exercise sheets for use of the feedback pen should be addressed to ensure that a new calibration is not necessary at the beginning of each exercise. Participants had no suggestions to improve the delivery of the cues or feedback.

## 4. Discussion

In the present study, the effects of continuous visual cueing and intermittent intelligent feedback on handwriting were compared for the first time. Results revealed that short-term training with both cueing and feedback can improve writing amplitude in both patients with PD and healthy controls for different writing amplitudes. Contrary to the immediate detrimental effects of visual cueing on writing at small amplitudes (0.6 cm) [17], the current study therefore suggests that the accuracy constraints of visual cueing can be overcome with proper training and that participants can learn how to use the cues to their advantage. As improvements were found for both the trained and untrained tasks and for both measurement tools, these results also suggest transfer of learning, in line with previous work [39]. Furthermore, amplitude improvements were found in the absence of cues or feedback. This is contrary to the guidance hypothesis of motor learning, stating that augmented sensory information during the acquisition phase of motor learning can cause dependency, leading to worse performance when cueing or feedback is withdrawn [19].

However, a strong tendency towards an interaction between training type and time for the large trained task depicts a more refined view. Patients and healthy controls did not deteriorate their writing amplitude during uncued tests after continuous visual cueing, but they did not improve either. On the other hand, after training with the feedback pen, amplitude increased, reflecting the absence of dependency and a possible advantage of training with an intermittent type of feedback [22]. Although the sample size was too small to draw definitive conclusions, the intelligent feedback likely forced participants to pay attention to specific aspects of the task, stimulating cognitive engagement and less habitual control [11, 41]. Future study in a larger sample needs to confirm whether this will lead to more robust learning. Also, training with the intelligent feedback pen could therefore be used to facilitate transfer of practice to daily life in more advanced stages of PD, which was shown to be more difficult in a previous study [42]. Another advantage of using the feedback pen is that is resembles writing in daily life better as it relies on pen and paper, rather than a tablet environment.

PD patients partaking in this study were all technology-literate and expressed an interest in undertaking a long-term training program using either the touch-sensitive tablet or the feedback pen. Both applications were well-tolerated and perceived as user-friendly tools by all participants, albeit that calibration procedures and pen grip will need further refinement. This points to the potential of both methods to serve as training tools for home use, offering the advantage that patients can practice fine motor skills without requiring transport to a rehabilitation clinic.

## 5. Limitations and Suggestions for Future Research

The current study has several limitations that may have influenced the outcomes. The most important drawback is the small sample size, which likely limited statistical power. As such, future studies should include larger sample sizes to investigate the specific benefit of intermittent intelligent feedback for patients with differences in disease severity. In this regard, the feedback pen has the additional advantage that it also allows gradual withdrawal of feedback, as the time between feedback messages can be easily altered. Though this was not applied in the current study, future research should investigate whether this approach can be used to further reduce cue- and feedback-dependency in PD [23]. Secondly, a systematic difference between performance with the pen and on the tablet was detected, indicating a tendency to write smaller with the pen. One possible explanation is that writing with the pen resembled more natural handwriting, as the typical friction between pen and paper is increased compared to the smoother surface of the touch-sensitive tablet [43, 44]. This may have led to better transfer at the expense of performance. In this regard, it would be interesting if future research could combine and compare different types of cueing and feedback delivery, that is, cued training on both a touch-sensitive tablet and on paper and feedback training on both paper and a touch-sensitive tablet.

## 6. Conclusion

In summary, the current study presented a novel feedback pen and compared it to visually cued writing training. The pen made it possible to receive personalized verbal feedback intermittently during writing practice. Online verbal corrections during writing practice proved to have a more robust beneficial learning effect than training supported by continuous visual cueing. This suggests that the feedback pen is a valuable tool to implement writing training in a tailor-made fashion for people with PD. As such, the findings are encouraging and future research should focus on including larger sample sizes and different subgroups of PD for long-term training.

## Figures and Tables

**Table 1:** General characteristics: median and interquartile ranges are displayed.

	Cue (*N* = 10)	Feedback (*N* = 9)	*p*-value
PD/CT	6/4	4/5	0.498
Age (years)	66.5 (55.0, 69.0)	52.0 (50.5, 68.5)	0.356
Gender (M/F)	6/4	4/5	0.498
Handedness (R/L)	9/1	7/2	0.842
MMSE (0–30)	28.5 (26.5, 30.0)	29.0 (28.5, 30.0)	0.447
MAM-16 (0–64)	60.0 (52.5, 63.3)	64.0 (61.0, 64.0)	0.065

PD specific			
Disease duration (years)	12.0 (7.3, 21.3)	5.5 (2.0, 9.0)	0.114
LED (mg/24 h)	740.0 (180.2, 1081.7)	482.5 (345.0, 515.0)	0.476
MDS-UPDRS-III (0–132)	35.5 (31.5, 44.0)	22.0 (13.3, 33.0)	0.067
NFOG-Q (0–24)	0.0 (0.0, 12.3)	0.0 (0.0, 9.0)	0.914

CT = healthy control; F = female; L = left; LED = Levodopa Equivalent Dose; M = male; MAM-16 = Manual Ability Measure; MMSE = Mini-Mental State Examination; R = right; MDS-UPDRS-III = Movement Disorder Society Unified Parkinson's Disease Rating Scale part III; PD = Parkinson's disease; NFOG-Q = New Freezing of Gait Questionnaire.
